# Nanoscale Spatial Control over the Self-Assembly of Small Molecule Hydrogelators

**DOI:** 10.3390/gels11040289

**Published:** 2025-04-14

**Authors:** Samahir Sheikh Idris, Hucheng Wang, Yuliang Gao, Peiwen Cai, Yiming Wang, Shicheng Zhao

**Affiliations:** State Key Laboratory of Chemical Engineering, Shanghai Key Laboratory of Multiphase Materials Chemical Engineering, School of Chemical Engineering, East China University of Science and Technology, Shanghai 200237, China; samahir694@gmail.com (S.S.I.); 15736220823@163.com (H.W.); y20210008@mail.ecust.edu.cn (Y.G.); peiwencai0512@163.com (P.C.)

**Keywords:** gels, small-molecular-weight-hydrogelators, gelation, directed self-assembly, supramolecular chemistry

## Abstract

Spatial control over molecular self-assembly at the nano scale offers great potential for many high-tech applications, yet remains a challenging task. Here, we report a polymer brush-mediated strategy to confine the self-assembly of hydrazone-based hydrogelators exclusively at nanoparticle surfaces. The surfaces of these nanoparticles are grafted with negatively charged polyacrylic acid, which enrich protons that can catalyze the in situ formation and self-assembly of hydrazone-based gelators. We found that, with respect to the polymer lengths, the concentration of the nanoparticles presents more significant effects on the self-assembly process and the properties of the resultant hydrogels, including gelation time, stiffness, and network morphology. More interestingly, the hydrogel fibers are found to be formed specifically around the nanoparticles, demonstrating the directed nanoscale molecular self-assembly. This work demonstrates that triggering molecular self-assembly using catalysis can serve as an effective way to realize directed molecular self-assembly at the nano scale, which may serve as a powerful approach to improve many material properties, such as the mechanical properties of supramolecular materials as we found in this work.

## 1. Introduction

Surface functionalization has become an effective approach to managing interactions between materials and their environments [1]. The recent boom in nanoarchitectonic systems opens new possibilities for creating chemical systems that are situated on surfaces and can respond to various stimuli from their environment [2]. This emerging discipline is deemed crucial in chemistry, as it enables the incorporation of innovative smart functionalities into materials for a range of applications. Starting from this perspective, directed molecular self-assembly has emerged as a prominent topic. Directed molecular self-assembly is widespread in nature, underpinning many pivotal biological functions and potential applications [3,4]. For instance, the collagen matrix is self-assembled in the presence of hydroxyapatite nanoparticles, providing structural integrity and strength to the bones [5,6]. As an inspiration, directed molecular self-assembly has garnered extensive interest because of its potential to give many interesting properties to supramolecular materials, for instance the enhancement of stiffness by enabling precise molecular organization and strong interactions at nanoparticle surfaces [7].

In this context, some supramolecular materials are formed through the self-assembly of low molecular weight building blocks, which have gained significant attention due to their dynamic, reversible, and stimuli responsive nature [8]. These supramolecular materials exhibit unique properties such as self-healing, adaptability, and tunable functionality [9], making them highly attractive for applications in fields such as nanotechnology, biomedicine, and energy storage [10]. The supramolecular material forms well-defined structures through noncovalent interactions, such as hydrogen bonding, π-π stacking, hydrophobic effects, and electrostatic interactions. These interactions allow the creation of materials that can adapt to the changes in their environment or enhance their different properties, making them ideal for different applications [11,12].

In recent years, various surface-assisted molecular self-assembly approaches have been built on these principles and developed to facilitate the growth of supramolecular materials at defined interfaces [13,14,15]. Basically, surface-assisted self-assembly is realized by triggering the self-assembly of molecular building blocks using stimuli such as protons [16,17,18,19], catalysts (enzyme) [20,21,22,23], and nucleation reagents [16,24,25,26]. For instance, Tait et al., gave an example of surface-assisted self-assembly, where Pentacene molecules self-assembled on the surface of metal substrate (Cu 100). This process is driven by the combination of interactions, including Van der Waals, electrostatic, hydrogen bonding, π-π stacking, and chemical bonding. Additionally, the incorporation of metal–organic coordination and substrate–molecule interactions plays a significant role in influencing the stability and overall structure of the assembly over longer distances. These interactions also enhance the two-dimensional confinement of the supramolecular material on the substrate surface [27]. Yang and colleagues studied the self-assembly of elastin-like peptides on pyrolytic graphite surfaces through the spatial confinement of fibril nucleation [28]. Another example by Datar et al. demonstrates a surface-assisted self-assembly approach to create well-defined and uniformed large areas of nanofibril supramolecular structures. This process is driven by the in situ self-assembly of an m-phenylene ethynylene macrocycle on a glass surface, facilitated primarily by π-π stacking [29]. Furthermore, Ciesielski’s group explored the supramolecular material formed through the self-assembly of oleylamine and the metallophthalocyanines on various surfaces, particularly graphene surfaces. These structures are created through non-covalent interactions, leading to the spontaneous formation of supramolecular lamellar arrangements and lattices. Their work highlights how the surface itself, along with specific characteristics, plays a critical role in assisting the self-assembly process. By providing a suitable environment, the surface enables the interaction and self-assembly necessary for applications in nanoelectronics [30]. Another group represents an innovative idea involving a bioactive seed layer that triggers the enzyme-assisted self-assembly of peptide fiber networks, especially at the film–solution interface [31]. Vigier-Carrière’s group studied the impact of surface properties and the self-assembly of low-molecular-weight hydrogelators through the “seed layer” on the surface to initiate gelation and self-assembling the hydrogelator molecules directly at the surface [7]. Additionally, Frisch developed a strategy to grow supramolecular copolymers on gold surfaces using oppositely charged peptide comonomers [32]. In a different approach, peptide-based low-molecular-weight hydrogels demonstrate the ability to grow not only on solid surfaces, as reported in the last study, but also in liquid environments, where their self-assembly can be precisely controlled. This process is driven by communication of interactions, including hydrogen bonding, hydrophobic effects, electrostatic forces, van der Waals forces, aromatic stacking, and water-mediated interactions [33,34]. An approach by Naydenove and his group investigated the self-assembly of tetraferrocene-porphyrin molecules on a gold surface, driven by hydrogen bonding, π-π stacking, and Van der Waals interactions. This group also emphasized the significance of tuning non-covalent interactions to achieve precise control over size and aggregation pattern for the resulting supramolecular material [35]. Spitzer and his group developed a method for surface-assisted self-assembly through proton diffusion, leading to the creation of supramolecular hydrogel structures at solid–liquid interfaces [36]. Dergham’s group reported the supramolecular self-assembly molecules on the surface of living cells through host–guest interaction, pH-induced assembly, and intracellular polymerization [37]. Lu et al. explored molecular self-assembly on the surfaces, focusing on the self-assembly of di-carbonitrile polyphenyls on metal surface. By integrating the ultra-high-vacuum (UHV) preparation method, the supramolecular nanostructures successfully dispersed across the metal surface. The self-assembly process was driven by a combination of non-covalent interactions. In addition, metal coordination bonds on metal surfaces also played a particularly critical role for this self-assembly stabilization [38]. In a related study, Li and his team investigated the manipulation and control of 10,12-pentacosadiynoic acid (PCDA) self-assembly on highly oriented pyrolytic graphite (HOPG) surface. Self-assembly was governed by more than four different interactions. These interactions included Van der Waals, electrostatic, hydrogen bonding, π-π stacking, and some chemical bonding. External factors such as substrate potential, presence of solvents, and confinement were found to significantly influence these interactions, enabling precise manipulation and control of the self-assembly process and the resultant supramolecular structure [39]. In recent years, Yao et al. used lattice-guided assembly of optoelectronically active π-conjugated materials to understand supramolecular self-assembly on inorganic 1D van der Waals crystals of NbS3 surfaces [40]. Despite these advances in spatially controlled self-assembly, directed molecular self-assembly at the nano scale remains a challenge [41,42], the realization of which may provide further solutions towards high-performance supramolecular materials and unlock many high-tech applications. The key obstacles lie in the competition between the self-assembly and spontaneous free diffusion of small molecules.

**Figure 1:**
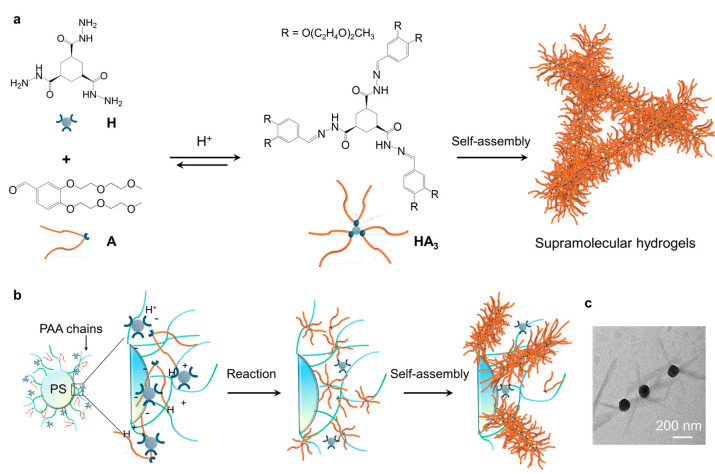
Concept of directed molecular self-assembly at the surfaces of nanoparticles: (**a**) scheme showing the catalytic formation and self-assembly of the hydrogelator **HA_3_**; (**b**) the formation and self-assembly of **HA_3_** around the catalytic nanoparticles; (**c**) TEM image showing the formation of hydrogel fibers around the nanoparticles.

In recent years, we and the van Esch group have systematically studied a hydrazone-based supramolecular hydrogelation system, where the gelator **HA_3_** is formed from the precursors hydrazide **H** and aldehyde **A** through the formation of hydrazone bonds (Figure 1a) [43]. By using catalysts, like acids or aniline, the formation and self-assembly of **HA_3_** can be dramatically accelerated. Here, we demonstrate a polymer brush-mediated strategy to achieve spatial control over the self-assembly of **HA_3_** at nanoparticle surfaces. Our system employs polyacrylic acid (PAA)-grafted polystyrene nanoparticles to create confined proton-rich microdomains that precisely localize both formation and self-assembly of **HA_3_** within nanometers of the particle surface (Figure 1b). The effects of different parameters of the nanoparticles, including the concentrations and brush length of the nanoparticles on the hydrogel formation and the properties of the resultant hydrogels were studied. Our results demonstrate that the concentration relative to brush length of the nanoparticles has a greater effect on the formation and the material properties of the hydrogels, such as gelation time, mechanical stiffness, and network structure. Importantly, the hydrogel fibers were found to be specifically formed around the nanoparticles, demonstrating the access to directed nanoscale molecular self-assembly (Figure 1c).

## 2. Results and Discussion

### 2.1. Characterization of the Prepared Nano-Sized Polyacrylic Acid Brushes

**Figure 2:**
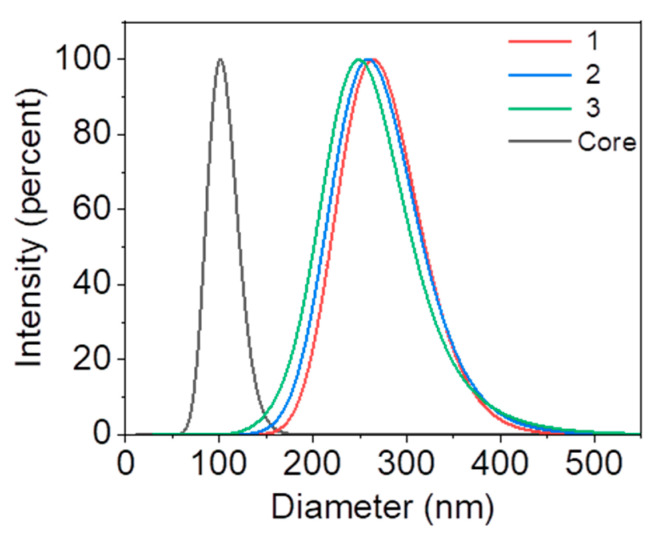
DLS results showing the dynamic hydration diameter of the nanoparticles grafted with negatively charged polymer chains of different lengths. All the nanoparticles grafted with PAA chains (Sample 1–3) are prepared using the same batch of core nanoparticles with a diameter of 96 nm.

In this study, nanoparticles with different brush lengths (156, 168, and 175 nm, respectively) were prepared by initiating the growth of polyacrylic acid (PAA) chains from the surface of polystyrene nanoparticles via surface-initiated photoemission polymerization [44]. Particle size distribution and zeta potential were measured using the dynamic light scattering (DLS) technique. The results show that the diameter of the core polystyrene nanoparticles is 96 ± 6 nm. In contrast, the overall diameters with polyacrylic acid brushes were measured with dimensions of 252 nm, 264 nm, and 271 nm, variations attributed to differing reaction times. Zeta potential assessments indicated that these nanoparticles possess a negatively charged surface, with potentials recorded at −7.5, −8.4, and −12.3 mV, respectively, as shown in Figure 2 and Table 1.

### 2.2. Effects of Brush Length and Concentration on the Self-Assembly and Properties of the Molecular Hydrogels

The impacts of nanoparticle concentrations and polymer brush length on gelation kinetics and mechanical stiffness of the resultant hydrogels were investigated by rheology. The samples were prepared by mixing the stock solutions of **H** and **A** at pH 7.0, and unless mentioned otherwise, the molar ratio of **H** and **A** was kept at 1:4 to ensure a complete conversion of **H** into **HA_3_**.

**Figure 3:**
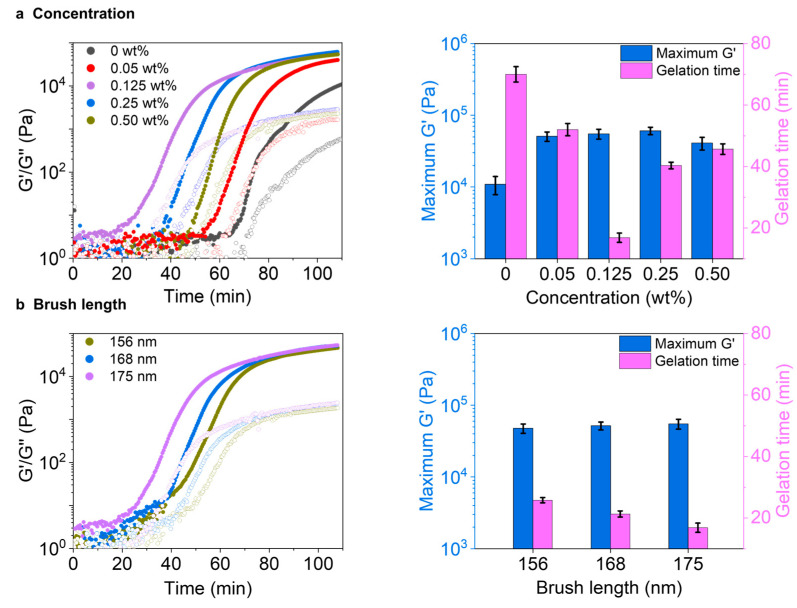
Rheological tests on the hydrogel formation in the presence of different nanoparticles. Effects of (**a**) nanoparticle concentration and (**b**) brush length on the hydrogelation process and the stiffness of the resultant hydrogels. All the samples: [**H**] = 20 mM, [**A**] = 80, at pH 7.0.

To study the effects of nanoparticle concentration on the gelation process, the hydrogel precursor solutions containing different concentrations of nanoparticle (from 0 to 0.5 wt%) were prepared and measured using an oscillatory rheometer. The brush length was kept at 175 nm. As shown in (Figure 3a), by increasing the nanoparticle concentration from 0 to 0.125wt%, the plateau storage modulus, G’, increases from 10 to 55 kPa. Further increase in the nanoparticle concentration does not lead to additional increase of G’. The higher G’ of the sample with the addition of more nanoparticles can be due to the higher catalysis, which leads to the rapid formation and self-assembly of the gelators, thereby resulting in a more branched hydrogel network [16,43,45,46,47]. Moreover, the gelation time (defined as the time at which the G’ reaches a plateau) decreases from 73 to 17 min by enhancing the concentration of nanoparticles from 0 to 0.125 wt%. However, interestingly, a further increase in the nanoparticle concentration from 0.125 to 0.5 wt% leads to an increase in the gelation time from 17 to 45 min (Figure 3a). We reason that the local formation of more hydrogel fibers around the nanoparticles causes the lower gelation rate at higher concentrations of nanoparticles. As such, fewer hydrogel fibers contribute to the formation of hydrogel networks, thereby leading to a longer gelation time. It should be noted that all the hydrogels are immune to the variation of oscillating frequency, indicating their stable solid gel state, and undergo a gel-to-sol transition when the strain is almost 10% (Figure S1).

Next, the effects of polymer brush lengths on the gelation were investigated by keeping the concentration of nanoparticles at a constant value of 0.125 wt%. The results show that by increasing the brush length from 156 to 175 nm, the longest brush length gives the shortest gelation time of 17 min and the highest G’ of 55 kPa compared to the shortest brush length values of 26 min and 47 kPa, respectively (Figure 3b). This higher G’ value of the sample with 175 nm can refer to the fiber formation and self-assembly of the gelators along the longest brush, resulting in relatively more branched hydrogel networks. The frequency sweep is crucial for understanding material behavior across different oscillation rates, which also follows the concentration manner to maintain a stable solid gel state and undergoes a gel-to-sol transition when the strain reaches 10%.

### 2.3. Effects of the Brush Concentration on the Network Morphologies of the Molecular Hydrogels

**Figure 4:**
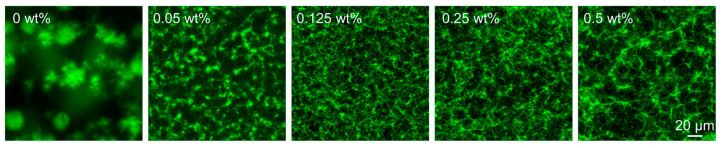
CLSM images showing the hydrogel networks formed in the presence of different concentrations of nanoparticles. Samples: [**H**] = 20 mM, [**A**] = 80, [**FITC**] = 30 µM at pH 7.0.

To learn more about the impact of nanoparticle concentrations and the length of polymer brushes on the hydrogel stiffness, we examined the morphologies of the resulting hydrogel networks through confocal laser scanning microscopy (CLSM). The hydrogels were formulated with a fluorescein aldehyde derivative (FITC) (Figure S2), resulting in fluorescently labeled networks. As shown in Figure 4, we observed that by increasing the concentration of the nanoparticles from 0 to 0.125 wt%, the resultant hydrogel networks evolved from large fibrous clusters to dense and relatively uniform fibrous networks. However, interestingly, with a further increase in the nanoparticle concentration, the network turned out to be looser. It seems that more hydrogel fibers are formed around the nanoparticles rather than growing towards the bulk area, thus leading to a looser network. The dependence of network morphologies on the nanoparticle concentration is in line with the above-discussed rheological results. In the samples prepared in the presence of nanoparticles with different brush lengths, the network morphologies are almost identical; no obvious differences are observed. Only in the magnified version, with the brush length increasing to 175 nm, the fibers tend to be a little denser than the shorter brush length of 156 nm (Figure S3). From the previous analysis, while ≈ 20 nm difference in the brush length may appear small, our rheological and morphological analysis demonstrates that this variation has only a marginal effect on the hydrogelation process and the material properties of the resultant hydrogels. Specifically, in different brush lengths, the gelation time varied by just ~9 min and stiffness by ~8 kPa, suggesting the system is far more sensitive to nanoparticle concentration, where the differences reach more than ~50 min and ~40 kPa. Again, here in CLSM images, the fiber network densities are nearly identical compared to the difference in the nanoparticle concentrations, confirming that the polymer brush length plays a secondary or tiny role in this system.

### 2.4. Location of the Self-Assembled Hydrogel Fibers with Respect to the Nano-Sized Polymer Brushes

**Figure 5:**
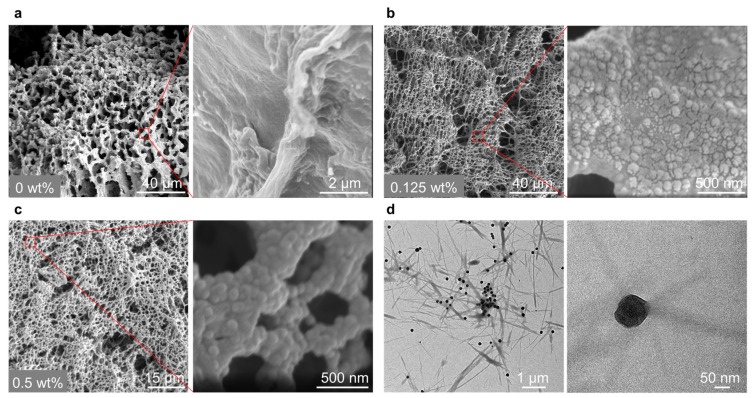
SEM pictures showing the hydrogel network morphology formed in the presence of different nanoparticle concentrations, (**a**–**c**) is 0, 0.125, and 0.5 wt%, respectively, insets are the magnified images. Samples: [**H**] = 20 mM, [**A**] = 80 at pH 7.0; and (**d**) TEM pictures demonstrating the localized formation of the hydrogel fibers in the vicinity of the nanoparticles. The sample: [**H**] = 5 mM, [**A**] = 20 at pH 7.0.

To gain further insight into the growth of the hydrogel fibers in the presence of catalytic nanoparticles, we employed scanning electron microscopy (SEM) to observe the distribution of nanoparticles in the hydrogel network. The SEM observations reveal that nanoparticle concentration significantly impacts the fiber structure (Figure S4). Without the catalyst, the fibers appear inhomogeneous, the fibers seem to collapse into large bundles (Figure 5a). However, at the optimal nanoparticle concentration of 0.125 wt%, dense fibrous networks are observed (Figure 5b). Upon magnifying the network, densely and uniformly packed nanoparticles are found to be distributed in the fibrous network. This explains the above-discussed high G’ and dense network morphology. Conversely, at a concentration of nanoparticles of 0.5 wt%, the network is looser relative to that of 0.125 wt% nanoparticles. Moreover, when we magnify the hydrogel network, a densely comprised nanoparticle network is observed (Figure 5c). The high concentration of nanoparticles likely leads to the formation of more hydrogel fibers around the nanoparticles, thereby generating a hydrogel network composed of aggregated nanoparticles rather than hydrogel fibers. This formation mechanism also explains the above-observed decreased G’ and looser hydrogel networks.

To delve deeper into the interaction between the fibers and the nanoparticles, we characterized the optimal sample using transmission electron microscopy (TEM). From TEM pictures, it is evident that the fibers selectively grow in the vicinity of the nanoparticles (Figure 5d and Figure S5). This result suggests that the nanoparticles function as catalysts to locally accelerate the formation and self-assembly of **HA_3_** around them.

## 3. Conclusions

In conclusion, we have leveraged varying lengths of acidic polymer grafted on nanoparticles to locally enable self-assembly of hydrazone-based gelators around the nanoparticle surface. Our results highlighted that the nanoparticle concentration relative to the brush length dominates the hydrogelation process and the material properties of the resultant hydrogels. At low concentration ranges, more nanoparticles lead to faster hydrogelation and a stiffer hydrogel with more branched fiber networks because of the higher catalysis. However, at the high concentration range, the variation of the corresponding properties with the nanoparticle concentration is reversed compared to that in the low concentration range because most of the hydrogel fibers are formed around the nanoparticles, thus limiting the crosslinking density of the fibrous networks. Microscopy observations at the nano scale have confirmed that the hydrogel fibers are specifically formed around the nanoparticles, demonstrating the spatially controlled molecular self-assembly at the nano scale. It should be noted that a 20 nm variation in brush length may not be sufficient to completely reveal the effect of brush length on the self-assembly process and the properties of the resultant hydrogels. However, the photoemulsive polymerization method that we used can only grow a less than 200 nm PAA brush, and with a shorter PAA brush with a length below 150 nm, the size uniformity of the PAA brush will be sacrificed. In the future, preparing PAA brushes with larger ranges of brush length are expected to address this pending issue. This work may contribute to the development of supramolecular materials with improved properties through directed nanoscale molecular self-assembly.

## 4. Materials and Methods

### 4.1. Materials

All chemicals used in this study were procured from Sigma-Aldrich (St. Louis, MO, USA). Molecules **H**, **A**, and the fluorescein aldehyde derivative (FITC) were synthesized following the established protocols documented in prior research [43]. Polyacrylic acid brushes incorporated in Polystyrene nanoparticles were synthesized in accordance with previously published work [48].

### 4.2. Concentration Measurement of the Prepared Particle Solutions

To determine the solid content of the nanoparticle solution, 1 mL of each synthesized nanoparticle solution was transferred into vials. The solvent was evaporated at 120 °C for three hours until the total weight of the vials remained constant. This solid content was calculated using the following formula:

$$\mathrm{Solid}\mathrm{content}\left(\%\right)=\frac{m2-mc}{m1-mc}\times 100\%\;$$

where *mc*, *m*1, and *m*2 represent the mass of the empty vial, the vial with the particles before drying, and the vial with the particles after drying, respectively.

### 4.3. Nanoparticle Size Distribution and Zeta Potential

Particle size distribution and zeta potential were measured using a Malvern Nano ZS 3600 Zetasizer (Malvern Instruments Ltd., Malvern, Worcestershire, UK). The particle concentrations for this measurement were standardized to 0.005 wt% in distilled water at pH 7.0. A laser wavelength of 633 nm and a scattering angle of 173° were employed. Measurement was conducted at 25 °C for 3 min, with each sample analyzed in triplicate [49].

### 4.4. Transmission Electron Microscopy

The morphology of hydrogelator cores and fiber was examined using a JEM 1400 model transmission electron microscope (TEM) (JEOL USA Inc., Jeol, Peabody, MA, USA). Samples were prepared by blending stock solutions of particles and gelator precursors to achieve a final concentration of 0.125 wt% catalysts, 5 mM of **H**, and 20 mM of **A**, adjusted to pH 7.0. The solution was serially diluted, and a droplet of the diluted solution was deposited onto copper grids. These grids were allowed to dry overnight at room temperature [48].

### 4.5. Rheological Analysis

Oscillatory rheology measurements were performed using a Themo HAAKE MARS 60 rheometer equipped with a 20 mm diameter stainless-steel parallel plate and a solvent trap to minimize solvent evaporation. Experiments were conducted in strain-controlled mode at 25 °C, with a strain amplitude of 0.05% and a frequency of 1.0 Hz. All samples were adjusted to pH 7.0 using distilled water. Stock solutions containing compounds **H**, **A**, and the catalyst were diluted to final concentrations of 20 mM for **H**, 80 mM for **A**, and varying catalyst concentrations (0, 0.05, 0.125, 0.25, and 0.5 wt%) to study the effect of catalyst concentration with a 175 nm brush length. For the catalyst brush length impact, the concentration was fixed at 0.125 wt%, with brush lengths of 156 nm, 168 nm, and 175 nm. After thoroughly mixing, the 150 µL samples were immediately transferred to the rheometer for measurement [43].

### 4.6. Confocal Laser Scanning Microscopy

Confocal microscopy was performed using a LEICA TCS SP8 confocal laser scanning system coupled with an Axio Observer inverted microscope and a 40x PlanFluor oil immersion objective lens (NA = 1.3). The fluorescein probe was excited using a 480 nm laser, and the pinhole was set to 1.0 airy unit to ensure optimal resolution. Data was analyzed using LASX imaging software. Samples were prepared by mixing solutions of **H** and **A** with the catalyst at concentrations of [H] = 20 mM, [**H**]:[**A**] = 1:4, pH 7.0, and a 30 μM fluorescent probe in distilled water. The samples were incubated overnight at room temperature in an imaging chamber before analysis [46].

### 4.7. Scanning Electron Microscopy

SEM imaging was conducted using a ZEISS Sigma 300 VP microscope (Carl ZEISS AG., Oberkochen, Germany) operating at 3 kV with a beam current of 5 µA, later adjusted to 2 µA. Samples were prepared by carefully depositing 200 µL of gel onto a glass coverslip. Ethanol was gradually added to dehydrate the samples, increasing the ethanol concentration by 10% every 10 min over one hour without agitation. The gel was then transferred to a coverslip and freeze-dried. To minimize charging effects and enhance imaging quality, samples were affixed to SEM stubs and sputter-coated with a thin layer of gold using a plasma coater [50].

## Figures and Tables

**Table 1:** Size and zeta potential measurements of the catalytic nanoparticles grafted with PAA chains.

Sample No	Core Size	Particle Size	Brush Length	Zeta Potential
1	96 nm	271 nm	175 nm	−12.3
2	96 nm	264 nm	168 nm	−8.4
3	96 nm	252 nm	156 nm	−7.5

## Data Availability

All data and materials are available upon request from the corresponding author.

## Supplementary Materials

The following supporting information can be downloaded at: https://www.mdpi.com/article/10.3390/gels11040289/s1, Figure S1: Rheological results show the effects of nanoparticle parameters on hydrogel formation. Effects of (a) nanoparticle concentration and (b) brush length on the hydrogelation process and the stiffness of the resultant hydrogels. All the samples: [H] = 20 mM, [A] = 80, at pH 7.0; Figure S2: Fluorescein aldehyde derivative (FITC); Figure S3: CLSM images showing the hydrogel networks formed in the presence of different brush lengths of nanoparticles. Samples: [H] = 20 mM, [A] = 80, [FITC] = 30 µM at pH 7.0; Figure S4: SEM pictures showing the hydrogel network morphology formed in the presence of different nanoparticle concentrations, (a–c) is 0, 0.125, and 0.5 wt%, respectively. Samples: [H] = 20 mM, [A] = 80 at pH 7.0; Figure S5. TEM pictures demonstrate the localized formation of the hydrogel fibers in the vicinity of the nanoparticles. The sample: [H] = 5 mM, [A] = 20 at pH 7.0.

## Abbreviations

The following abbreviations are used in this manuscript:

HA3	Hydrazone Gelators
H	Hydrazide
A	Aldehyde
PAA	Polyacrylic Acid
TEM	Transmission Electron Microscopy
CLSM	Confocal Laser Scanning Microscopy
FITC	Fluorescein Aldehyde Derivative
SEM	Scanning Electron Microscopy
