# Supplementary material for: Nanoscale Spatial Control over the Self-Assembly of Small Molecule Hydrogelators
Source: Gels. 2025 Apr 14;11(4):289. doi: 10.3390/gels11040289 (PMC12026908; doi:10.3390/gels11040289)
Supplement: Supplementary file 1 [file gels-11-00289-s001.zip › gels-3537975-supplementary.pdf]

Supporting Information

Nanoscale Spatial Control over the Self-Assembly of Small Molecule Hydrogelators

Samahir Sheikh Idris, Hucheng Wang, Yuliang Gao, Peiwen Cai, Yiming Wang*, Shicheng Zhao*

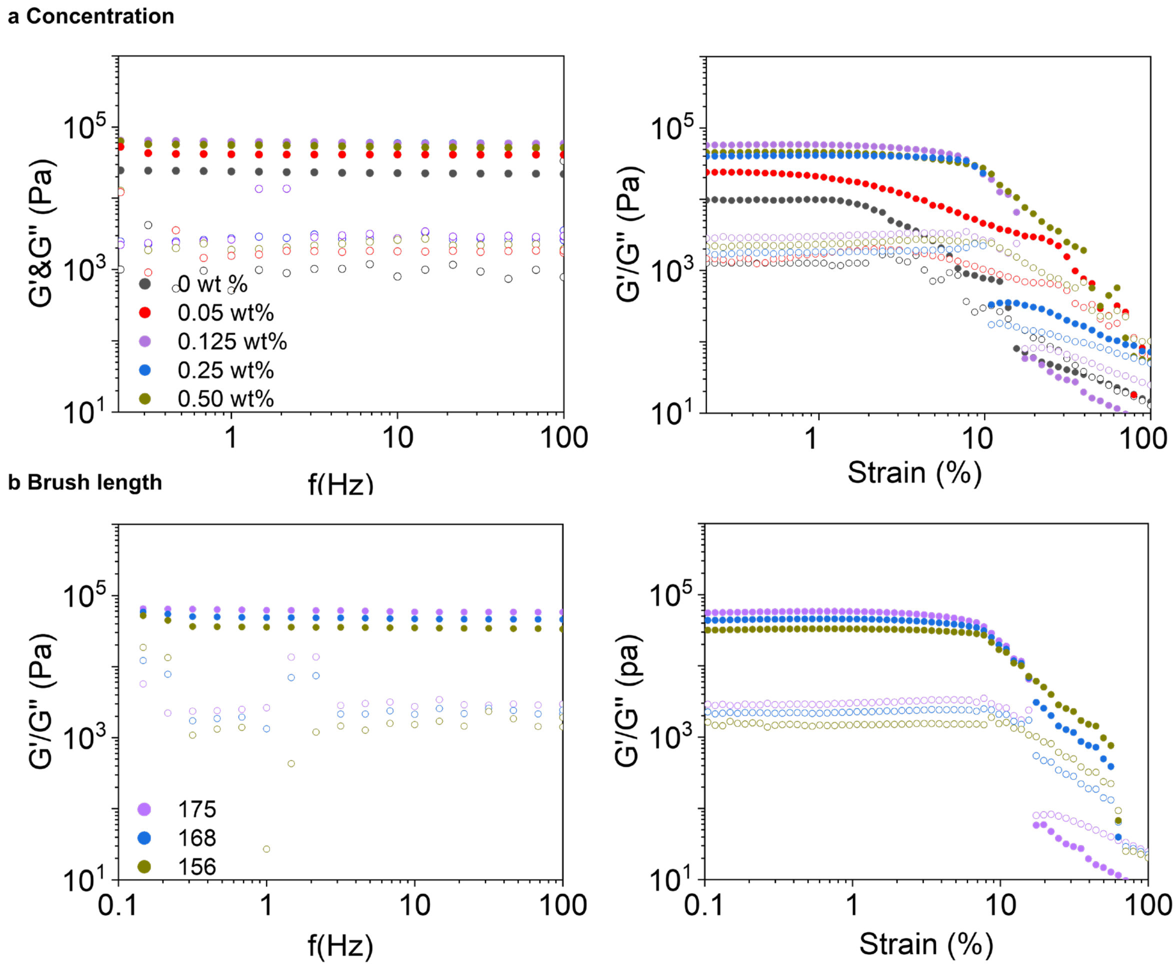

Figure S1: Rheological results show the effects of nanoparticle parameters on hydrogel formation. Effects of (a) nanoparticle concentration and (b) brush length on the hydrogelation process and the stiffness of the resultant hydrogels. All the samples: [**H**] = 20 mM, [**A**] = 80mM, at pH 7.0.

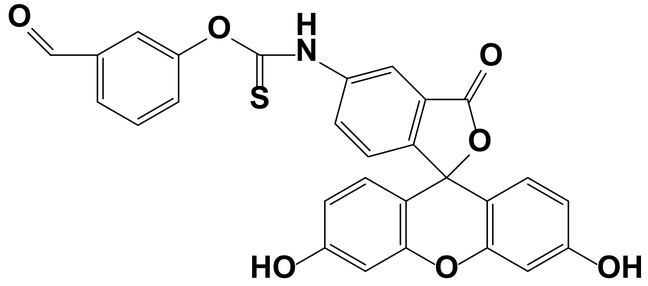

Figure S2: Fluorescein aldehyde derivative (FITC)

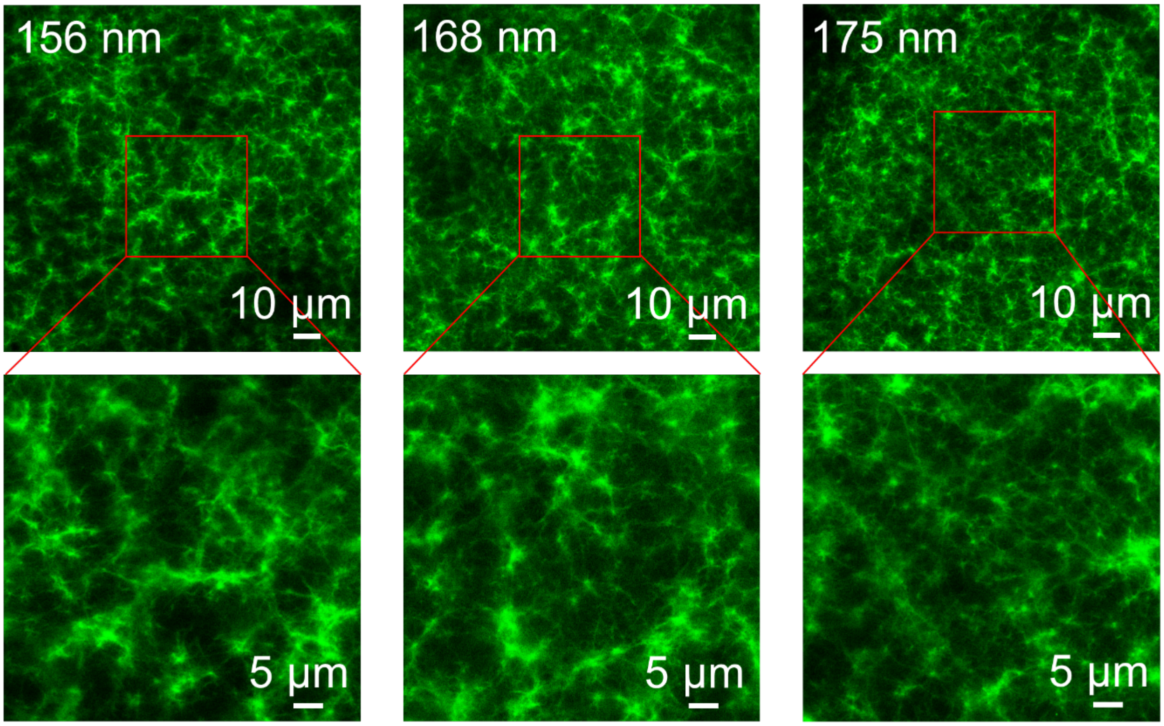

Figure S3. CLSM images showing the hydrogel networks formed in the presence of different brush lengths of nanoparticles at 0.125 wt%. Samples: [**H**] = 20 mM, [**A**] = 80mM, [**FITC**] = 30 µM, at pH 7.0.

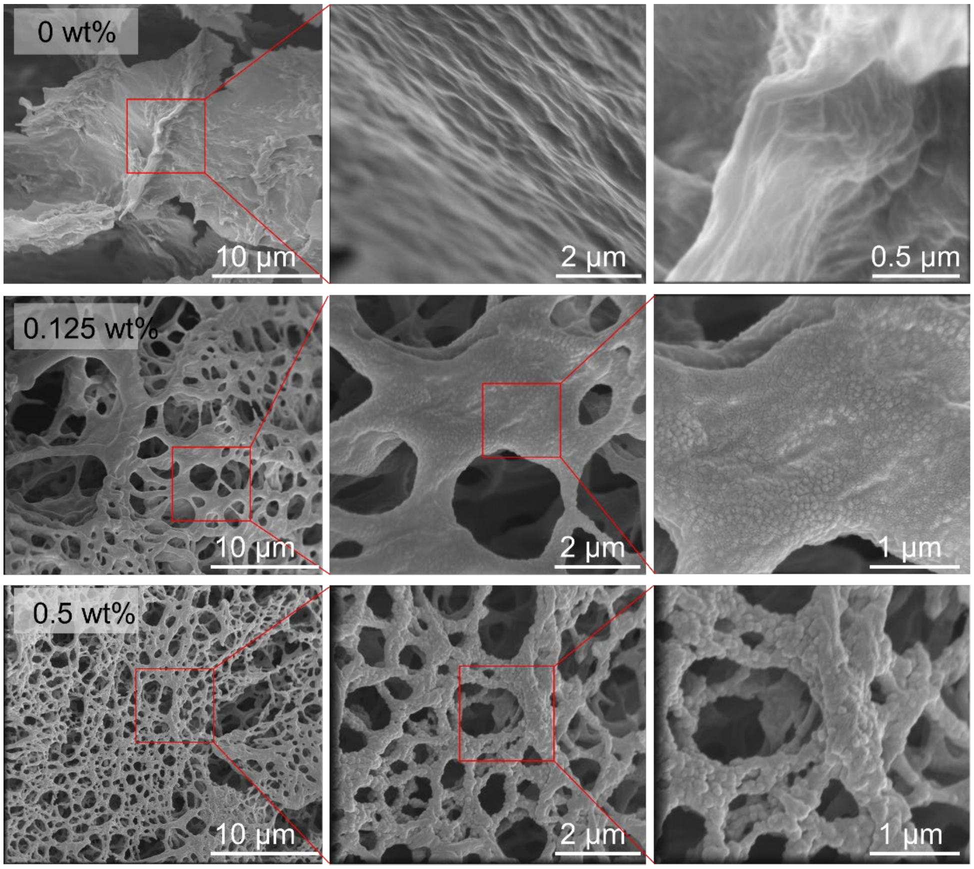

Figure S4. SEM pictures showing the hydrogel network morphology formed in the presence of different nanoparticle concentrations of 0, 0.125, and 0.5 wt%, respectively. Samples: [**H**] = 20 mM, [**A**] = 80mM at pH 7.0.

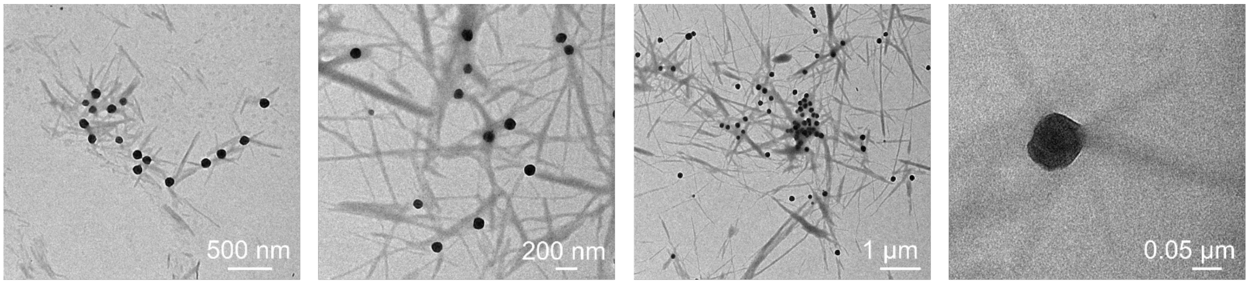

Figure S5. TEM pictures demonstrate the localized formation of the hydrogel fibers in the vicinity of the nanoparticles. The sample: [**H**] = 5 mM, [**A**] = 20mM at pH 7.0.
